# Supplementary material for: The LOTUS domain of Oskar promotes localisation of both protein and mRNA components of Drosophila germ plasm
Source: bioRxiv. 2025 May 6:2025.05.02.651258. Preprint. [Version 1] doi: 10.1101/2025.05.02.651258 (PMC12247748; doi:10.1101/2025.05.02.651258)
Supplement: Supplement 1 [file media-1.pdf]

Supplemental Information for

The LOTUS domain of Oskar promotes localization of both protein and mRNA components of *Drosophila* germ plasm

Anastasia Repouliou, John R. Srouji, Emily L. Rivard, Andrés E. Leschziner, Cassandra G. Extavour

This Supplemental Information contains the following elements:

- Supplementary Figure S1 through S4
- Supplementary Tables S1 through S5
- Key Reagents Table

SUPPLEMENTAL FIGURES AND LEGENDS

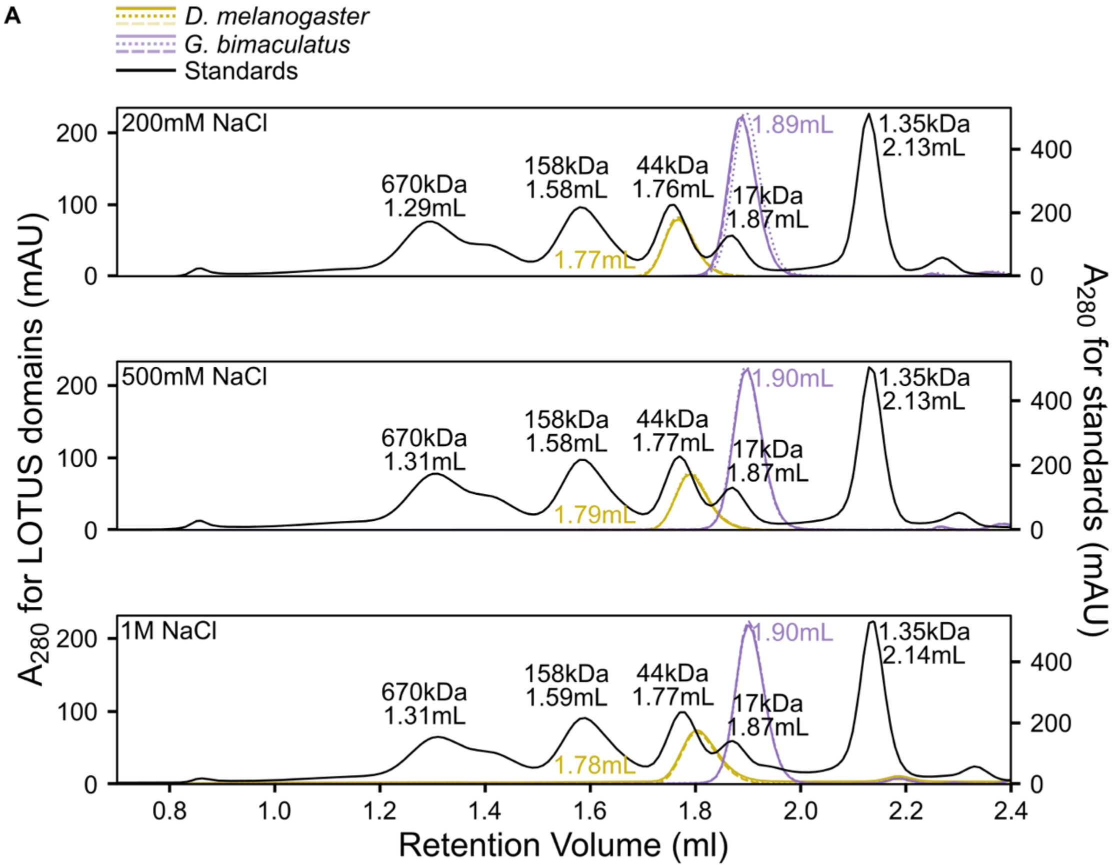

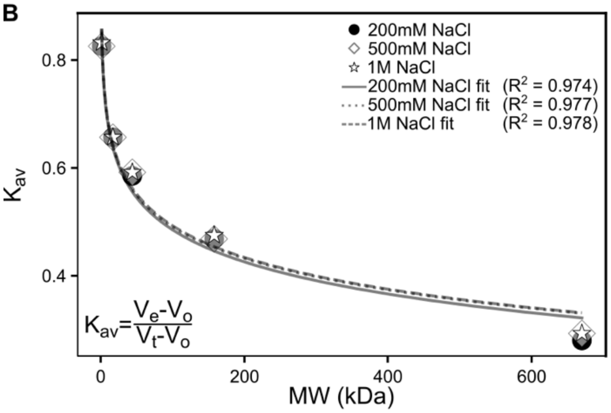

C

[NaCl] (Mm)	*D. melanogaster* apparent MW (kDa) FW 12.3kDa	*G. bimaculatus* apparent MW (kDa) FW 10.5kDa
200	28.8	11.7
500	26.0	11.2
1000	25.6	11.4

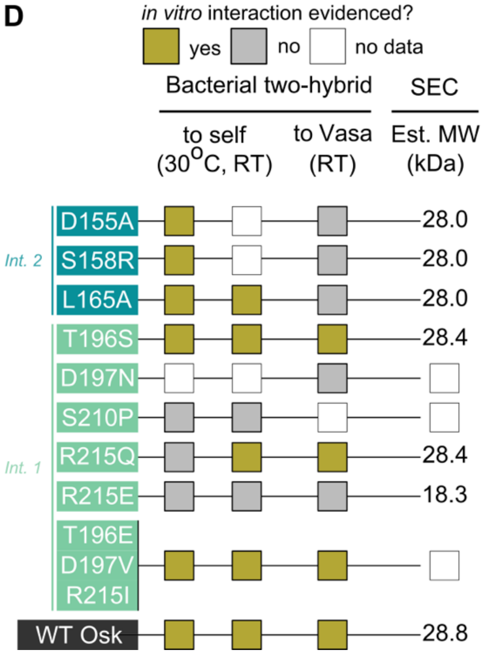

Figure S1. *D. melanogaster* Oskar LOTUS behaves as a dimer and *G. bimaculatus* Oskar LOTUS as a monomer *in vitro*. (B) Overlaid A280 chromatograms from triplicate gel filtration experiments of *D. melanogaster* Oskar LOTUS domain (gold curves; formula weight 12.3 kDa) and *G. bimaculatus* Oskar LOTUS domain (purple curves; formula weight 10.5 kDa) with standards (black curve; corresponding to bovine thyroglobulin (670 kDa), bovine gamma-globulin (158 kDa), chicken ovalbumin (44 kDa), horse myoglobin (17 kDa), and vitamin B12 (1.35 kDa)) performed with 20mM Tris pH 8.0 and either 200mM, 500mM, or 1M NaCl running buffer. Solid, dotted, and dashed lines represent independent runs. The monomeric molecular weights and peak retention volumes of standards and samples are indicated. All runs were performed on a Superose 6 PC 3.2/30 column (GE Healthcare). (**B**) Calibration curves for each analytical SEC buffer condition, generated by fitting the gel filtration standards data in (A). See Materials & Methods for full description of the quantification. (**C**) The apparent molecular weights of the *D. melanogaster* and *G. bimaculatus* Oskar LOTUS domains were determined by comparing their observed partition coefficients (see Materials & Methods) with the corresponding standards calibration curve in (B). The formula weight (FW) for each monomeric protein is indicated. **(D)** Bacterial two-hybrid assay (BACTH) at 30◦C and room temperature (RT) evidencing *in vitro* interaction (gold square), lack of *in vitro* interaction (grey squares), or no data (white squares). D197N, S210P, and T196E-D197V-R215I were insoluble. Analytical SEC used to calculate estimated molecular weight (Est. MW).

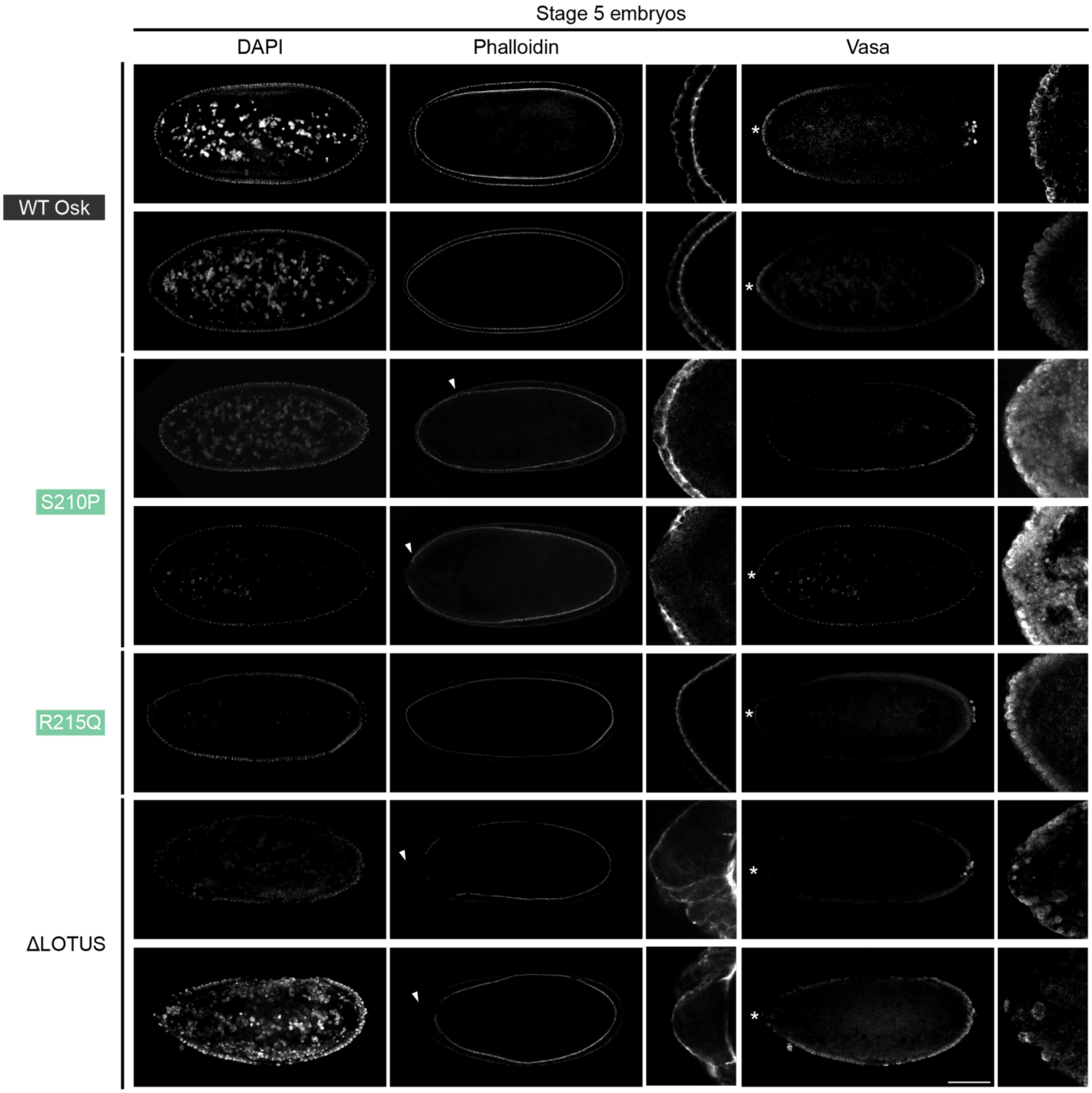

Figure S2. Examples of anterior pole cell attempts. Optical sections and magnified insets of confocal micrographs of stage 5 embryos from *y^1 v^1/w^*; $P\{y^+$ v^+ = (oskar variant-HA-bcd3'UTR)\}attp40/+; $P\{w^{+mC}$=matalpha4-GAL-VP16\}V37/+* mothers. Fixed embryos were stained for nuclei (DAPI), F-actin (phalloidin) for staging, the HA epitope (anti-HA antibody), and Vasa (anti-Vasa antibody) to mark pole cells. Anterior is to the left. The R215Q and last ΔLOTUS embryos were masked for visualization purposes. Micrographs were selected to show examples of phenotypes scored as developmental defects (arrowheads, shown at higher magnification to the right) and/or anterior pole cell attempts (asterisks, shown at higher magnification to the right). See Table S2 for full scoring table. Scale bar = 100μm and applies to all panels.

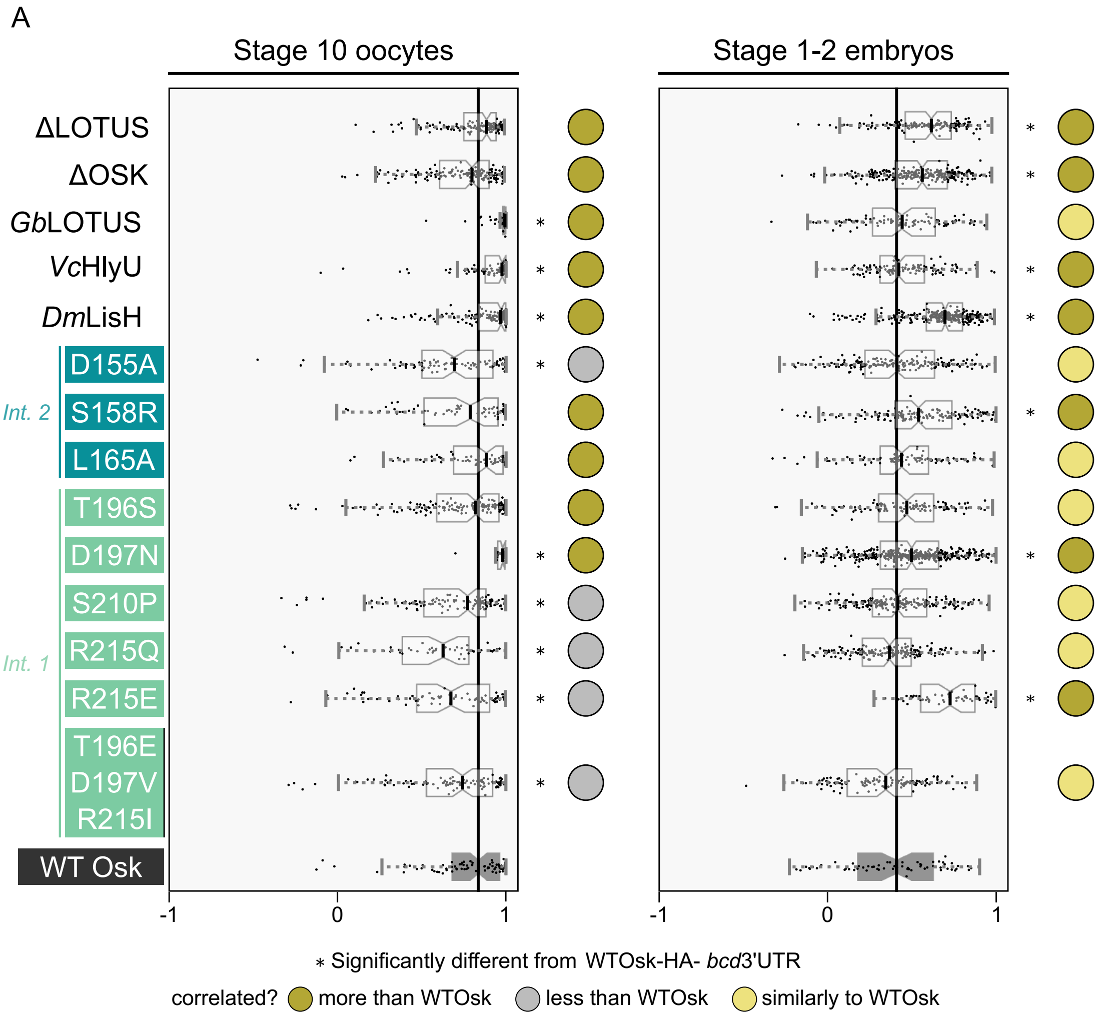
A
Stage 10 oocytes
Stage 1-2 embryos
ΔLOTUS
ΔOSK
GbLOTUS
VcHlyU
DmLisH
D155A
S158R
L165A
Int. 2
T196S
D197N
S210P
R215Q
R215E
T196E
D197V
R215I
Int. 1
WT Osk
-1
0
1
* Significantly different from WTOsk-HA- bcd3'UTR
correlated? more than WTOsk less than WTOsk similarly to WTOsk

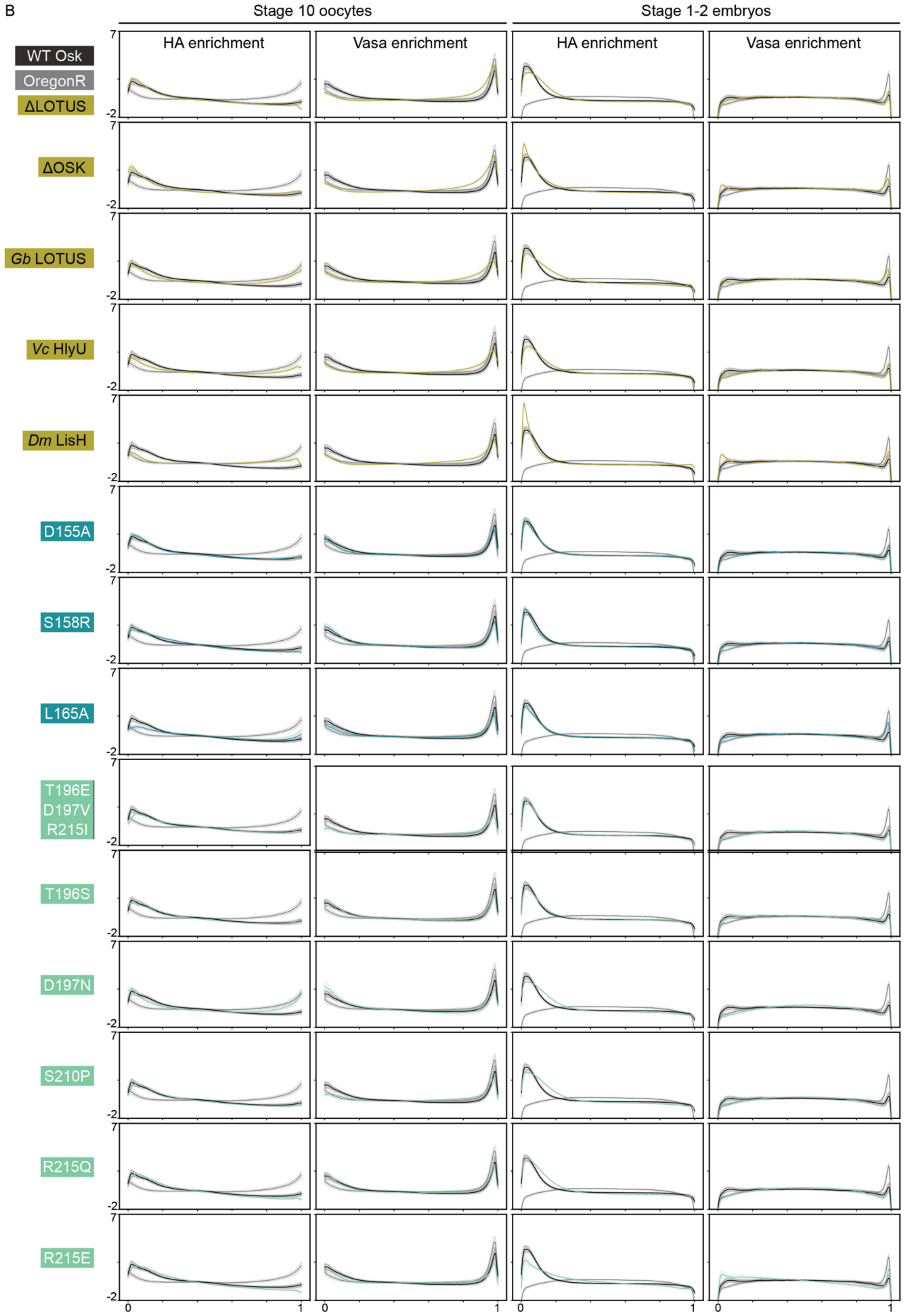

B
Stage 10 oocytes
Stage 1-2 embryos
HA enrichment
Vasa enrichment
HA enrichment
Vasa enrichment
WT Osk
OregonR
ΔLOTUS
ΔOSK
Gb LOTUS
Vc HlyU
Dm LisH
D155A
S158R
L165A
T196E D197V R215I
T196S
D197N
S210P
R215Q
R215E
7
-2
0
1

Figure S3. Pearson correlation of HA-Vasa signal intensity suggests Oskar-Vasa anterior colocalisation in LOTUS variants. (A) Box plots of the Pearson correlation between the HA and Vasa signal in the anterior pixels with the highest HA intensity values (with intensity values above the threshold of mean + 2 standard deviations of HA intensity values for each micrograph). Stage 10 oocytes (left) and stage 1-2 embryos (right), *y^1 v^1/w^*; P{y^+ v^+ = (wt-osk-HA-bcd3'UTR)}attp40/+; P{w^{+mC}=matalpha4-GAL-VP16}V37/+* (positive control) in black, median as solid black line. Significant difference from positive control distribution (p-value < 0.05, estimated using a bootstrapped test (10,000 iterations)) indicated by asterisk. Circles indicate data interpretation: HA-Vasa correlation in variant significantly more than (dark gold), less than (grey), or not significantly different from (light gold) positive control. (**B**) HA and Vasa signal enrichment (z-score) across normalised anteroposterior axis (anterior at 0, posterior at 1) in stage 10 oocytes (left) and stage 1-2 embryos (right). Mean (solid line) and 95% confidence intervals (shaded regions) shown, Oregon R (negative control) in gray, *y^1 v^1/w^*; P{y^+ v^+ = (wt-osk-HA-bcd3'UTR)}attp40/+; P{w^{+mC}=matalpha4-GAL-VP16}V37/+* (positive control) in black.

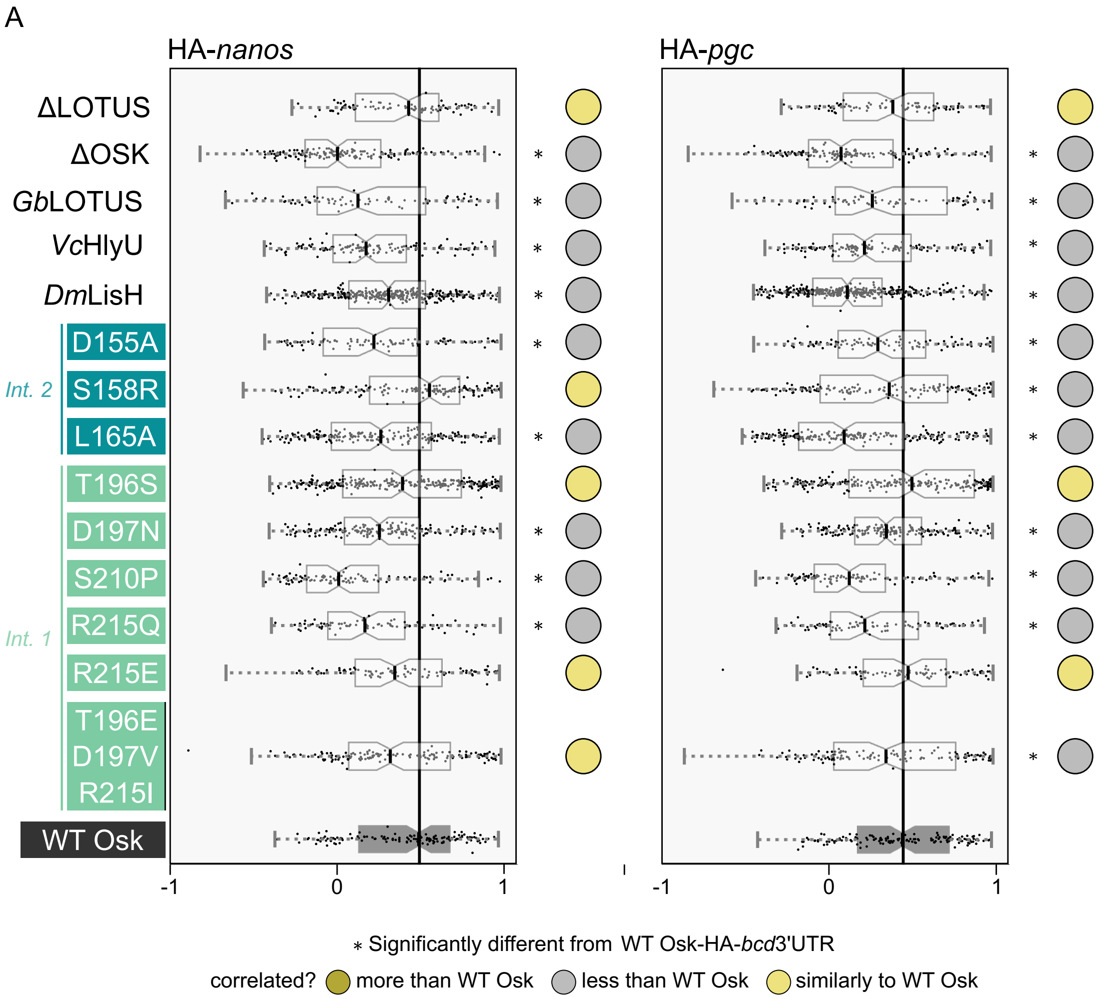
A
HA-nanos
HA-pgc
ΔLOTUS
ΔOSK
GbLOTUS
VcHlyU
DmLisH
Int. 2
D155A
S158R
L165A
Int. 1
T196S
D197N
S210P
R215Q
R215E
T196E
D197V
R215I
WT Osk
-1
0
1
* Significantly different from WT Osk-HA-bcd3'UTR
correlated? more than WT Osk less than WT Osk similarly to WT Osk

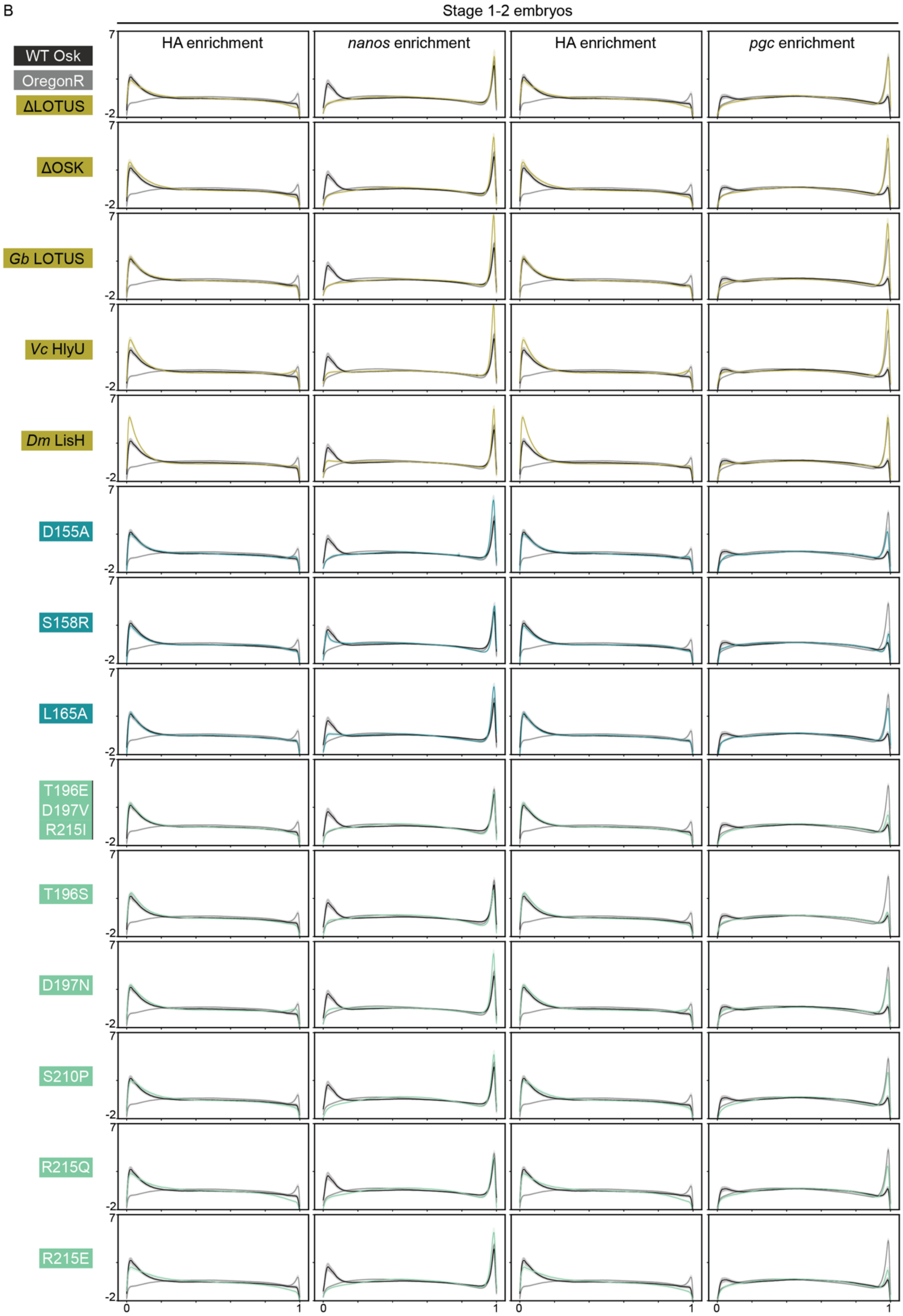
B
Stage 1-2 embryos
HA enrichment
nanos enrichment
HA enrichment
pgc enrichment
WT Osk
OregonR
ΔLOTUS
ΔOSK
Gb LOTUS
Vc HlyU
Dm LisH
D155A
S158R
L165A
T196E
D197V
R215I
T196S
D197N
S210P
R215Q
R215E
7
-2
0
1

Figure S4. Pearson correlation of HA-*nanos* and HA-*pgc* signal intensity suggests Oskar-mRNA anterior colocalisation in LOTUS variants. (A) Box plots of the Pearson correlation between the HA and *nanos* (left) and *pgc* (right) signals in the anterior pixels with the highest HA intensity values (with intensity values above the threshold of mean + 2 standard deviations of HA intensity values for each micrograph). *y^1 v^1/w^*; P{y^+ v^+ = (wt-osk-HA-bcd3'UTR)}attp40/+; P{w^{+mC}=matalpha4-GAL-VP16}V37/+* (positive control) in black, median as solid black line ar. Significant difference from positive control distribution (p-value < 0.05, estimated using a bootstrapped test (10,000 iterations)) indicated by asterisk. Circles indicate interpretation of data: HA-mRNA correlation in variant significantly more than (dark gold), less than (grey), or not significantly different from (light gold) *y^1 v^1/w^*; P{y^+ v^+ = (wt-osk-HA-bcd3'UTR)}attp40/+; P{w^{+mC}=matalpha4-GAL-VP16}V37/+*. (**B**) HA and *nanos* (left) and HA and *pgc* (right) signal enrichment (z-score) across normalized anteroposterior axis (anterior at 0, posterior at 1) in stage 1-2 embryos. Mean (solid line) and 95% confidence intervals (shaded regions) shown, Oregon R (negative control) in gray, *y^1 v^1/w^*; P{y^+ v^+ = (wt-osk-HA-bcd3'UTR)}attp40/+; P{w^{+mC}=matalpha4-GAL-VP16}V37/+* (positive control) in black.

88

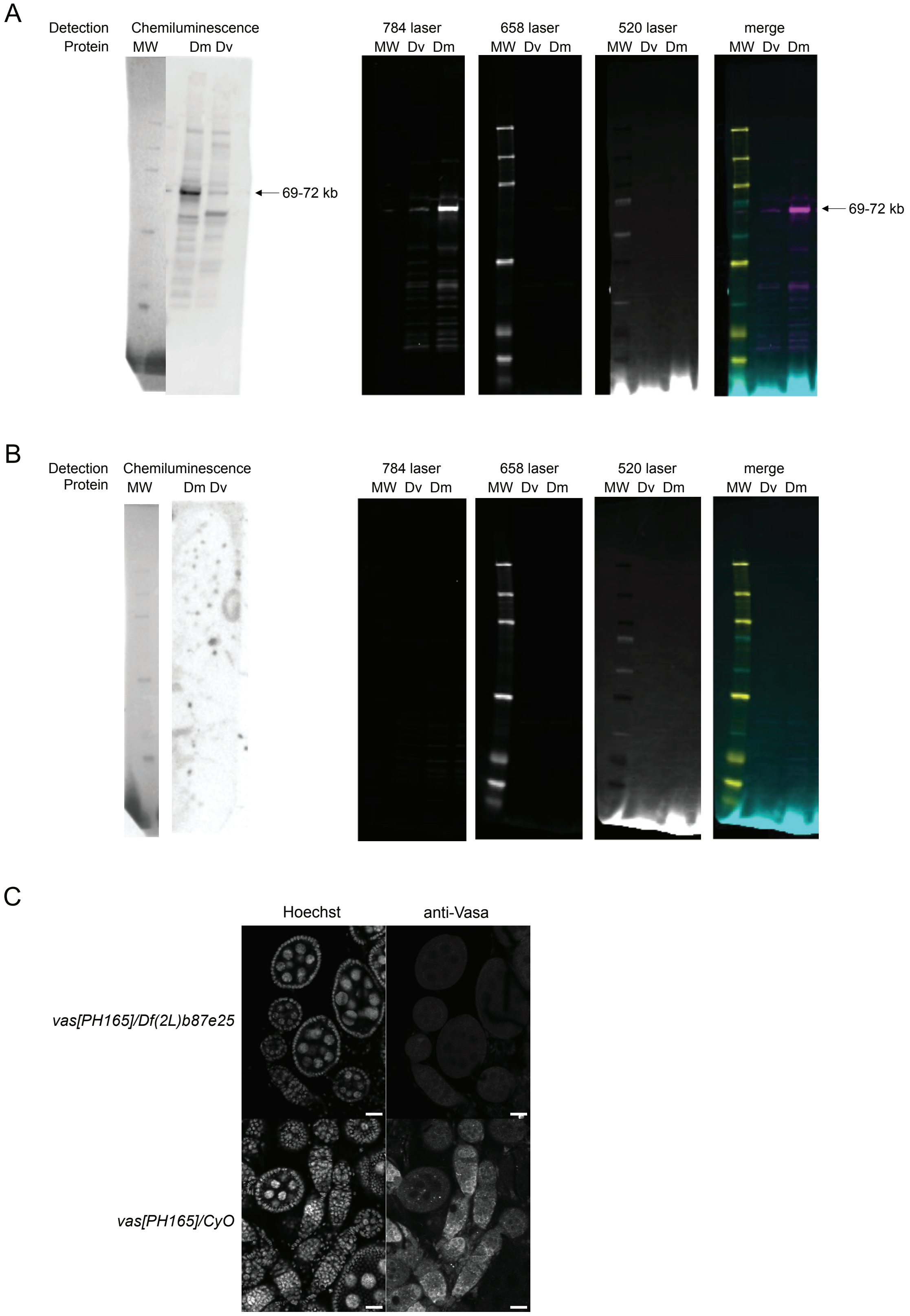

A
Detection
Protein
Chemiluminescence
MW Dm Dv
69-72 kb
784 laser
MW Dv Dm
658 laser
MW Dv Dm
520 laser
MW Dv Dm
merge
MW Dv Dm
69-72 kb
B
Detection
Protein
Chemiluminescence
MW Dm Dv
784 laser
MW Dv Dm
658 laser
MW Dv Dm
520 laser
MW Dv Dm
merge
MW Dv Dm
C
Hoechst
anti-Vasa
vas[PH165]/Df(2L)b87e25
vas[PH165]/CyO

89

Figure S5. *D. melanogaster* anti-Vasa antibody validation. (**A–B)** Chemiluminescent (left) and fluorescent (right) Western Blots with *D. melanogaster* (Dm) and *D. virilis* (Dv) ovary lysates. Lanes with molecular weights are marked MW. Chemiluminescent Western blots were imaged using a CCD camera on an Azure Sapphire Biomolecular Imager. Fluorescent blots were excited by three laser lines and captured using APD detectors on an Azure Sapphire Biomolecular Imager. Signal from the primary antibody is excited by the 784 nm laser, while the molecular weight is excited by the 658 nm and 520 nm lasers. Bands at expected product sizes are marked with an arrow (*D. melanogaster* Vasa ~72 kb, *D. virilis* Vasa ~69kb). Blots were incubated in anti-*D. melanogaster* Vasa (A) or no primary antibody (B). **(C)** Vasa antibody staining in *D. melanogaster* ovaries in flies with genotypes *vas[PH165]/Df(2L)b87e25* (*vasa* null) and *vas[PH165]/CyO* (control). Images are single optical sections and scale bar represents 20 µm.

SUPPLEMENTAL TABLES AND LEGENDS

Table S1. Summary of diffraction data for Losk139-241 crystal forms 1–4

**Crystal Form**	**1**	**2**	**3**	**4**
**Sample**	Losk139-241	Losk139-241	Losk139-241	Losk139-241 + $HgCl_2$
**Space Group**	P4	I4	C222	$P12_11$
**a, b, c (Å)**	138.95, 138.95, 61.90	141.1, 141.1, 219.9	128.6, 205.4, 172.4	39.60, 63.57, 40.15
**α, β, γ (º)**	90, 90, 90	90, 90, 90	90, 90, 90	90, 97.22, 90
**Wavelength (Å)**	0.9800	0.9800	0.9800	1.0039
**No. of unique reflections**	18398	20643	18656	13341
**Resolution Range (Å)**	50 – 3.21	50 – 3.13	50 – 4.16	50 – 2.00
**R_{merge}**	0.053	0.107	0.158	0.198
**Overall I/σ(I)**	8.0 (1.2)	9.6 (1.3)	5.4 (1.1)	16.0 (1.9)
**Completeness (%)**	92.6	99.0	98.4	100.0 (100.0)
**Multiplicity**	1,8	3.5	2.8	16.3
**Anomalous completeness (%)**	-	-	-	99.9 (99.9)
**Anomalous multiplicity**	-	-	-	8.2

Table S2. Summary of stage 5 embryo analysis for presence of anterior pole cells.

				HA-positive[a]			HA-negative[b]		
**Variant**	**Embryos[c]**	**Stage 5[d]**	**HA positive[e]**	**Developmental defect[f]**	**Anterior pole cell attempt[g]**	**Anterior pole cells[h]**	**Developmental defect[f]**	**Anterior pole cell attempt[g]**	**Anterior pole cells[h]**
**Oregon R**	347	298	4	2	0	0	1	0	0
**ΔLOTUS**	549	524	314	4	2	3	14	5	1
**ΔOSK**	222	217	216	23	24	0	1	1	0
**Gb LOTUS**	424	412	148	6	6	0	35	28	0
**Vc HlyU**	703	696	172	3	0	0	12	21	0
**Dm LisH**	443	436	372	36	11	0	16	8	0
**D155A**	920	874	307	28	0	0	16	1	2
**S158R**	501	469	363	8	1	6	2	0	0
**L165A**	742	689	379	44	0	2	33	0	0
**T196S**	601	586	384	42	4	8	10	0	1
**D197N**	586	549	307	24	2	1	34	0	0
**S210P**	853	799	355	48	2	2	32	2	8
**R215Q**	885	836	439	19	10	0	9	2	0
**R215E**	1482	1376	118	17	0	0	92	0	3
**Triple[*]**	427	417	200	2	2	0	3	1	0
**WT Osk**	648	625	238	14	26	36	9	60	21

[a] Analysis of stage 5 embryos with anterior HA enrichment.
[b] Analysis of stage 5 embryos without anterior HA enrichment, indicating either absence of HA-tagged Oskar variant at the anterior or inefficient HA staining.
[c] Number of embryos imaged per variant. See Materials & Methods for collection, staining, and imaging protocol.
[d] Number of imaged embryos visually determined to be stage 5 based on phalloidin stain showing completed cellularisation but not gastrulation.
[e] Number of stage 5 embryos visually determined to show anterior enrichment of HA signal, indicating anterior enrichment of HA-tagged Oskar variant.
[f] Number of stage 5 embryos visually determined to show developmental defects. Developmental defect phenotypes include uneven cellularisation around the periphery of the embryo and cellular budding reminiscent of pole cell budding at ectopic (non-posterior or anterior) locations. See Fig. SX for examples.
[g] Number of stage 5 embryos visually determined to show anterior PC attempts based on nuclear (DAPI) and Vasa stain. PC attempts defined as budding, high Vasa signal, or spherical nuclei in cells at the anterior, but with no fully budded PCs clearly identifiable. See Fig. 4A and Fig. S3 for examples.
[h] Number of stage 5 embryos visually determined to show anterior PCs based on nuclear (DAPI) and Vasa stain. For variants with a nonzero number of embryos with anterior pole cells, posterior and anterior pole cells counted for embryos with anterior pole cells (Fig. 4C).
[*] Triple refers to the triple mutant T196E-D197V-R215I.

Table S3. Statistical Analysis of anterior pole cell incidence and counts.

Variant	N[a]	Anterior pole cells[b]	p-value[c] (anterior pole cell incidence vs. Negative Control)	p-value[c] (anterior pole cell incidence vs. Positive Control)	p-value[d] (posterior pole cell count vs. Negative Control)	p-value[d] (anterior ole cell count vs. Positive Control)
**Oregon R**	298	0		4.144835e-14		0
**ΔLOTUS**	314	3	0.134433	3.000754e-11	0.10813455	0.47913206
**ΔOSK**	216	0	1	2.043245e-11		
**Gb LOTUS**	148	0	1	9.115716e-09		
**Vc HlyU**	172	0	1	9.388045e-10		
**Dm LisH**	372	0	1	3.395834e-16		
**D155A**	307	0	1	2.230220e-14		
**S158R**	363	6	0.026920	2.353295e-10	2.91486379e-09	0.00855579
**L165A**	379	2	0.313037	6.843532e-14	0	0.38321883
**T196S**	384	8	0.009781	1.093258e-09	1.89603991e-05	0.09808461
**D197N**	307	1	0.507438	5.065534e-13	1.11022302e-16	0.00531489
**S210P**	355	2	0.295169	2.850252e-13	0.00326256	5.44009282e-15
**R215Q**	439	0	1	7.170191e-18		
**R215E**	118	0	1	1.943480e-07		
**Triple[*]**	200	0	1	7.843703e-11		
**WT Osk**	238	36	4.144835e-14		2.25375274e-14	

[*] Triple refers to the triple mutant T196E-D197V-R215I.
[a] Number of stage 5 embryos (Oregon R) or number of stage 5 embryos with anterior HA enrichment.
[b] Number of embryos visually determined to show anterior PCs.
[c] p-value (calculated using Fisher's exact test, based on a 2×2 contingency table; exact probabilities are computed without relying on large-sample approximations).
[d] p-value (estimated using a bootstrapped test (10,000 iterations), comparing the observed difference in means from control distribution to a distribution generated by resampling with replacement.

Table S4. Statistical Analysis of Vasa enrichment in oocytes and embryos.								
	Stage 10 Oocytes				Stage 1-2 embryos			
**Variant**	**Count[a]**	**p-value[b] (Integrated Anterior Enrichment vs. Positive Control)**	**p-value[b] (Integrated Anterior Enrichment vs. Negative Control)**	**p-value[b] (Pearson Correlation vs. Positive Control)**	**Count[a]**	**p-value[b] (Integrated Anterior Enrichment vs. Positive Control)[b]**	**p-value[b] (Integrated Anterior Enrichment vs. Negative Control)**	**p-value[b] (Pearson Correlation vs. Positive Control)**
**Oregon R**	84	6.55018591e-07			189	0.00124249		
**ΔLOTUS**	138	5.89546013e-17	0.04460279	0.08094359	160	0.14182322	0.00535144	1.02738993e-06
**ΔOSK**	150	2.03899506e-18	0.02280415	0.10769372	265	0.38275709	9.17341758e-11	5.73561552e-05
**Gb LOTUS**	78	1.08245031e-08	0.33320252	2.21267449e-13	100	0.05963263	0.0152061	0.12304134
**Vc HlyU**	109	9.95357718e-13	0.19365831	1.91191839e-05	122	0.01947077	0.13663505	0.04612047
**Dm LisH**	182	5.56884297e-17	0.05777246	7.75637362e-05	307	0.18007473	1.73749903e-13	1.15651932e-12
**D155A**	97	0.01140579	0.00051423	0.00258703	191	0.01958909	0.11748532	0.19789815
**S158R**	66	0.02609067	3.78562737e-11	0.07952136	154	0.13775126	0.00311394	0.00022194
**L165A**	69	0.01265417	0.0076116	0.20661084	92	0.20000143	0.00187369	0.1183661
**T196S**	150	0.11665846	6.63691324e-13	0.09257797	118	0.48573134	0.00014762	0.09177468
**D197N**	31	0.0148029	8.1539997e-11	3.55437901e-12	409	3.68680593e-06	0.00531154	0.006517
**S210P**	126	1.95206258e-06	0.13607942	0.00450611	210	0.00954832	0.20107368	0.18511517
**R215Q**	50	0.13839737	1.93235927e-07	4.85141466e-05	179	0.00010435	0.10580469	0.20384861
**R215E**	73	0.01924888	0.00041599	0.00084778	58	0.00206767	7.92366173e-13	1.78301818e-13
**Triple[*]**	105	0.00014175	0.12874218	0.00619258	127	0.00437658	0.41212536	0.07212177
**WT Osk**	85		7.8202681e-07		65		0.00141613	

[*] Triple refers to the triple mutant T196E-D197V-R215I.
[a] Number of oocytes or embryos visually determined to show anterior enrichment of HA signal, indicating anterior enrichment of HA-tagged Oskar variant.
[b] p-value (difference from control distribution, 10,000 Simulations, bootstrapping with resampling)

Table S5. Statistical Analysis of *nanos* and *pgc* enrichment in stage 1-2 embryos.								
	nanos				***pgc***			
**Variant**	**Count[a]**	**p-value[b] (Integrated Anterior Enrichment vs. Positive Control)**	**p-value[b] (Integrated Anterior Enrichment vs. Negative Control)**	**p-value[b] (Pearson Correlation vs. Positive Control)**	**Count[a]**	**p-value[b] (Integrated Anterior Enrichment vs. Positive Control)[b]**	**p-value[b] (Integrated Anterior Enrichment vs. Negative Control)**	**p-value[b] (Pearson Correlation vs. Positive Control)**
**Oregon R**	723	1.51552666e-31			592	4.4135902e-08		
**ΔLOTUS**	85	3.17469149e-20	0.03501306	0.28582801	85	2.72834324e-17	3.19958328e-10	0.06045782
**ΔOSK**	150	2.34761833e-44	4.77285821e-09	6.3090931e-20	150	1.68497567e-16	6.31120934e-11	1.51666976e-15
**Gb LOTUS**	73	5.10266219e-37	0.00027754	1.2854597e-05	73	0.00382874	0.05428115	0.00657417
**Vc HlyU**	130	1.90779859e-18	5.4366558e-07	4.89546054e-07	130	0.1235291	4.02455846e-13	3.09157442e-06
**Dm LisH**	362	1.54521935e-09	1.22124533e-15	0.00050271	362	0.00668283	5.3265015e-05	1.63376578e-21
**D155A**	90	1.51706518e-27	0.30760755	0.00014871	90	5.15788935e-05	0.41437526	0.01011794
**S158R**	158	0.01816407	0.	0.08821007	156	0.00486035	0.00503855	0.00912196
**L165A**	215	1.41928405e-09	1.76503256e-12	0.00011704	215	1.02274157e-07	0.25075921	5.50798673e-15
**T196S**	289	1.2330628e-27	0.27089413	0.2882354	245	2.48909122e-09	0.04313497	0.26528852
**D197N**	173	2.73857579e-12	4.85779973e-08	0.00011259	170	0.19026173	6.25437357e-09	0.01049373
**S210P**	98	1.29226955e-60	1.24171548e-24	6.43372641e-13	98	2.15858897e-22	1.0067283e-18	1.04211795e-09
**R215Q**	79	2.43610099e-42	1.68498198e-09	1.06647914e-06	79	6.69470709e-12	3.97562825e-05	0.00013527
**R215E**	88	2.46350993e-49	1.5330683e-13	0.17277567	85	1.92576858e-06	0.12558829	0.36006112
**Triple[*]**	144	7.71456903e-30	0.03886315	0.11417878	125	7.20415414e-18	1.43887471e-11	0.019827
**WT Osk**	142		0.		137		5.62784721e-08	

[*] Triple refers to the triple mutant T196E-D197V-R215I.

[a] Number of oocytes or embryos visually determined to show anterior enrichment of HA signal, indicating anterior enrichment of HA-tagged Oskar variant.

[b] p-value (Difference from Control Distribution, 10,000 Simulations, bootstrapping with resampling)

KEY RESOURCES TABLE

REAGENT or RESOURCE	SOURCE	IDENTIFIER
**Antibodies**		
Mouse anti HA-Tag monoclonal antibody	ABclonal	AE008
Rat anti-HA High Affinity monoclonal antibody	Roche	3F10
Rabbit anti Vasa polyclonal antibody	Laboratory of Prashanth Rangan (Icahn School of Medicine at Mount Sinai)	
Chicken anti Vasa polyclonal antibody	This paper	9276
DAPI, for nucleic acid staining	Sigma-Aldrich	D9542
Rhodamine Phalloidin	Thermo Fisher Scientific	R415
Goat anti-Mouse IgG (H+L) Cross-Adsorbed Secondary Antibody, Alexa Fluor™ 488	Thermo Fisher Scientific	A-11001
Goat anti-Rat IgG (H+L) Cross-Adsorbed Secondary Antibody, Alexa Fluor™ 488	Thermo Fisher Scientific	A-11006
Goat anti-Rabbit IgG (H+L) Highly Cross-Adsorbed Secondary Antibody, Alexa Fluor™ 647	Thermo Fisher Scientific	A-21245
Goat anti-Chicken IgY (H+L) Cross-Adsorbed Secondary Antibody, Alexa Fluor™ 633	Thermo Fisher Scientific	A-21103
HCR™ RNA-FISH anti-nanos probe (v3.0)	Molecular Instruments	
HCR™ RNA-FISH anti-pgc probe (v3.0)	Molecular Instruments	
HCR™ RNA-FISH amplifier B5-555 (v3.0)	Molecular Instruments	
HCR™ RNA-FISH amplifier B4-647 (v3.0)	Molecular Instruments	
**Bacterial and virus strains**		
BL21(DE3) *E. coli*		
BTH101 *E. coli*	Euromedex	EUK001
DHM1 *E. coli*	Euromedex	EUK001
**Chemicals, peptides, and recombinant proteins**		

2-mercaptoethanol, Omnipur	Avantor	Cat# EM-6050
5x Pierce Lane Marker Non-Reducing Sample Buffer	Thermo Scientific	Cat# 39001
Precision Plus Protein Dual Color Standards	Bio-Rad	Cat# 1610394
Precision Plus Protein WesternC Blotting Standards	Bio-Rad	Cat# 1610376
SuperSignal West Pico PLUS Chemiluminescent Substrate	Thermo Scientific	Cat# 34580
Sodium azide	Sigma-Aldrich	Cat# S2002
Fluorescent Blot Blocking Buffer	Azure	Cat# AC2190
Azure Blot Washing Buffer	Azure	Cat# AC2113
Drosophila melanogaster Vasa (UniProt ID: M9PBB5, M1-E206 aa)	This paper	N/A
Ni-NTA Agarose	QIAGEN or Thermo Scientific	
Gel Filtration Standards	BIO-RAD	
Amicon Ultracel Devices	Millipore	
**Critical commercial assays**		
Pierce Detergent Compatible Bradford Assay Kit	Thermo Scientific	Cat# 23246
Trans-Blot Turbo RTA Mini 0.2 µm PVDF Transfer Kit	Bio-Rad	Cat# 1704272
Bacterial Adenylate Cyclase-based Two-Hybrid (BACTH) kit	Euromedex	EUK001
**Deposited data used in analyses**		
Raw and analysed data	This paper	
Oskar protein sequence *D. melanogaster*	UniProt	Acc. No. P25158
Oskar protein sequence *D. sechellia*	UniProt	Acc. No. B4HKZ1
Oskar protein sequence *D. simulans*	UniProt	Acc. No. B4QXC8
Oskar protein sequence *D. yakuba*	UniProt	Acc. No.

		B4PTX6
Oskar protein sequence *D. immigrans*	UniProt	Acc. No. A1Y1T7
Oskar protein sequence *D. virilis*	UniProt	Acc. No. Q24741
Oskar protein sequence *D. pseudoobscura*	UniProt	Acc. No. Q295Q4
Oskar protein sequence *D. erecta*	UniProt	Acc. No. B3P1W4
Oskar protein sequence *D. ananassae*	UniProt	Acc. No. B3LZ06
Oskar protein sequence *D. persimilis*	UniProt	Acc. No. B4GFV0
Oskar protein sequence *D. willistoni*	UniProt	Acc. No. B4N815
Oskar protein sequence *D. mojavensis*	UniProt	Acc. No. B4K9E4
Oskar protein sequence *D. grimshawi*	UniProt	Acc. No. B4JTJ1
Oskar protein sequence *Gryllus bimaculatus*	UniProt	Acc. No. K4MTL4
Vibrio cholera transcription factor HlyU	UniProt	Acc. No. C3LST3
D. melanogaster lis1 LisH domain	UniProt	Acc. No. Q7KNS3
Crystal structure of the LOTUS domain (aa 139-222) of *D. melanogaster* Oskar in C222	[1]	RCSB PDB 5A49
Crystal structure of the NTD L199M of *D. melanogaster* Oskar protein	[2]	RCSB PDB 5CD7
Structure of the LOTUS domain of *D. melanogaster* Oskar in complex with the C-terminal RecA-like domain of *D. melanogaster* Vasa	[3]	RCSB PDB 5NT7
**Experimental models: *D. melanogaster* strains**		
w[*]; P{w[+mC]=matalpha4-GAL-VP16}V37	Bloomington Drosophila	7063

	Stock Center	
y[1] v[1]; Sco/CyO	Laboratory of Norbert Perrimon lab (Harvard Medical School)	N/A
y[1] v[1]; attP40	Genetic Services, Inc.	
y[1] v[1]; P{y[+] v[+]=(wt-osk-HA-bcd3'UTR)}attp40	This paper	WT Osk
y[1] v[1]; P{y[+]v[+]=(Δ140-241-osk-HA-bcd3'UTR)}attp40	This paper	ΔLOTUS
y[1] v[1]; P{y[+]v[+]=(Δ401-606-osk-HA-bcd3'UTR)}attp40	This paper	ΔOSK
y[1] v[1]; P{y[+]v[+]=(Gbosk2-90-Dmosk-HA-bcd3'UTR)}attp40	This paper	Gb LOTUS
y[1] v[1]; P{y[+]v[+]=(VcHlyU2-109-Dmosk-HA-bcd3'UTR)}attp40	This paper	Vc HlyU
y[1] v[1]; P{y[+]v[+]=(DmLis2-86-Dmosk-HA-bcd3'UTR)}attp40	This paper	Dm LisH
y[1] v[1]; P{y[+]v[+]=(Dmosk-D155A-HA-bcd3UTR)}attp40	This paper	D155A
y[1] v[1]; P{y[+] v[+]=(Dmosk-S158R-HA-bcd3UTR)}attp40	This paper	S158R
y[1] v[1]; P{y[+]v[+]=(Dmosk-L165A-HA-bcd3UTR)}attp40	This paper	L165A
y[1] v[1]; P{y[+] v[+]=(Dmosk-T196S-HA-bcd3UTR)}attp40	This paper	T196S
y[1] v[1]; P{y[+] v[+]=(Dmosk-D197N-HA-bcd3UTR)}attp40	This paper	D197N
y[1] v[1]; P{y[+] v[+]=(Dmosk-S210P-HA-bcd3UTR)}attp40	This paper	S210P
y[1] v[1]; P{y[+] v[+]=(Dmosk-R215Q-HA-bcd3UTR)}attp40	This paper	R215Q
y[1] v[1]; P{y[+] v[+]=(Dmosk-R215E-HA-bcd3UTR)}attp40	This paper	R215E
y[1] v[1]; P{y[+]v[+]=(Dmosk-T196E-D197V-R215I-HA-bcd3UTR)}attp40	This paper	T196E

vas[PH165]/CyO; Drop/TM3	Laboratory of Paul Lasko (McGill University)	N/A
Df(2L)b87e25/CyO	Bloomington Drosophila Stock Center	BDSC 3138
**Recombinant DNA**		
pET-43.1b(+) expression vector	Novagen	
pET-Losk139-241_C10H	This paper	N/A
pET-151-Gbosk1-440	[4]	N/A
pET-151-Gbosk1-90	This paper	N/A
D. melanogaster transgenic vector pVALIUM22	Perrimon lab (Harvard Medical School)	N/A
pVAL22-Dmosk-bcd3UTR	This paper	N/A
pVAL22-Dmosk-HA-bcd3UTR	This paper	N/A
pVAL22-Dmosk-Δ140-241-osk-HA-bcd3UTR	This paper	N/A
pVAL22-Dmosk-Δ401-606-HA-bcd3UTR	This paper	N/A
pVAL22-Gbosk2-90-Dmosk-HA-bcd3UTR	This paper	N/A
pVAL22-VcHlyU2-109-Dmosk-HA-bcd3UTR	This paper	N/A
pVAL22-DmLis2-86-Dmosk-HA-bcd3UTR	This paper	N/A
pVAL22-Dmosk-D155A-HA-bcd3UTR	This paper	N/A
pVAL22-Dmosk-S158R-HA-bcd3UTR	This paper	N/A
pVAL22-Dmosk-L165A-HA-bcd3UTR	This paper	N/A
pVAL22-Dmosk-T196S-HA-bcd3UTR	This paper	N/A
pVAL22-Dmosk-D197N-HA-bcd3UTR	This paper	N/A
pVAL22-Dmosk-S210P-HA-bcd3UTR	This paper	N/A
pVAL22-Dmosk-R215Q-HA-bcd3UTR	This paper	N/A
pVAL22-Dmosk-R215E-HA-bcd3UTR	This paper	N/A
pVAL22-Dmosk-T196E-D197V-R215I-HA-bcd3UTR	This paper	N/A

Vibrio cholera transcription factor HlyU (residues 1 to 109)	DNASU Plasmid Repository	
pKNT25	Euromedex	EUK001
pKT25	Euromedex	EUK001
pUT18	Euromedex	EUK001
pUT18C	Euromedex	EUK001
**Software and algorithms**		
MAFFT	[5]	
PHENIX software package	[6–9]	
MODELLER	[10]	
HHpred webserver	[11]	
COOT	[12]	
PyMOL	[13]	
UNICORN software	GE Healthcare	
FIJI	[14]	
Python	Python Software Foundation	
FIJI macro code for microscopy image processing	This paper	https://github.com/Anastasiarep/LOTUS_mutant_analysis.git (commit ID 0de8b5a)
Python code for data analysis and figure generation	This paper	https://github.com/Anastasiarep/LOTUS_mutant_analysis.git (commit ID 0de8b5a)
Inkscape	Inkscape Project	

SUPPLEMENTAL REFERENCES

1. Jeske, M., Bordi, M., Glatt, S., Müller, S., Rybin, V., Müller, C.W., and Ephrussi, A. (2015). The crystal structure of the Drosophila germline inducer Oskar identifies two domains with distinct Vasa helicase- and RNA-binding activities. Cell Rep. *12*, 587–598. https://doi.org/10.1016/j.celrep.2015.06.055.

2. Yang, N., Yu, Z., Hu, M., Wang, M., Lehmann, R., and Xu, R.-M. (2015). Structure of Drosophila Oskar reveals a novel RNA binding protein. Proc Natl Acad Sci U S A *112*, 11541–11546. https://doi.org/10.1073/pnas.1515568112.

3. Jeske, M., Müller, C.W., and Ephrussi, A. (2017). The LOTUS domain is a conserved DEAD-box RNA helicase regulator essential for the recruitment of Vasa to the germ plasm and nuage. Genes Dev. *31*, 939–952. https://doi.org/10.1101/gad.297051.117.

4. Ewen-Campen, B., Srouji, J.R., Schwager, E.E., and Extavour, C.G. (2012). oskar Predates the Evolution of Germ Plasm in Insects. Curr Biol *22*, 2278–2283. https://doi.org/10.1016/j.cub.2012.10.019.

5. Katoh, K., and Standley, D.M. (2013). MAFFT Multiple Sequence Alignment Software Version 7: Improvements in Performance and Usability. Mol. Biol. Evol. *30*, 772–780. https://doi.org/10.1093/molbev/mst010.

6. McCoy, A.J. (2006). Solving structures of protein complexes by molecular replacement with Phaser. Acta Crystallogr. Sect. D, Biol. Crystallogr. *63*, 32–41. https://doi.org/10.1107/s0907444906045975.

7. McCoy, A.J., Grosse-Kunstleve, R.W., Adams, P.D., Winn, M.D., Storoni, L.C., and Read, R.J. (2007). Phaser crystallographic software. J. Appl. Crystallogr. *40*, 658–674. https://doi.org/10.1107/s0021889807021206.

8. Adams, P.D., Grosse-Kunstleve, R.W., Hung, L., Ioerger, T.R., McCoy, A.J., Moriarty, N.W., Read, R.J., Sacchettini, J.C., Sauter, N.K., and Terwilliger, T.C. (2002). PHENIX: building new software for automated crystallographic structure determination. Acta Crystallogr. Sect. D *58*, 1948–1954. https://doi.org/10.1107/s0907444902016657.

9. Adams, P.D., Gopal, K., Grosse-Kunstleve, R.W., Hung, L., Ioerger, T.R., McCoy, A.J., Moriarty, N.W., Pai, R.K., Read, R.J., Romo, T.D., et al. (2004). Recent developments in the PHENIX software for automated crystallographic structure determination. J. Synchrotron Radiat. *11*, 53–55. https://doi.org/10.1107/s0909049503024130.

10. Webb, B., and Sali, A. (2016). Comparative Protein Structure Modeling Using MODELLER. Curr. Protoc. Bioinform. *54*, 5.6.1-5.6.37. https://doi.org/10.1002/cpbi.3.

11. Söding, J., Biegert, A., and Lupas, A.N. (2005). The HHpred interactive server for protein homology detection and structure prediction. Nucleic Acids Res *33*, W244–W248. https://doi.org/10.1093/nar/gki408.

12. Emsley, P., and Cowtan, K. (2004). Coot: model-building tools for molecular graphics. Acta Crystallogr. Sect. D: Biol. Crystallogr. *60*, 2126–2132. https://doi.org/10.1107/s0907444904019158.

13. Schrödinger, and LLC The PyMOL Molecular Graphics System, Version 3.0.

14. Schindelin, J., Arganda-Carreras, I., Frise, E., Kaynig, V., Longair, M., Pietzsch, T., Preibisch, S., Rueden, C., Saalfeld, S., Schmid, B., et al. (2012). Fiji: an open-source platform for biological-image analysis. Nat Methods *9*, 676–682. https://doi.org/10.1038/nmeth.2019.
